# Probing Optical Nonlinearities of Unconventional Glass Nanocomposites Made of Ionic Liquid Crystals and Bimetallic Nanoparticles

**DOI:** 10.3390/nano12060924

**Published:** 2022-03-11

**Authors:** Valentyn Rudenko, Anatolii Tolochko, Svitlana Bugaychuk, Dmytro Zhulai, Gertruda Klimusheva, Galina Yaremchuk, Tatyana Mirnaya, Yuriy Garbovskiy

**Affiliations:** 1 Institute of Physics of National Academy of Sciences of Ukraine, 03680 Kyiv, Ukraine; val@iop.kiev.ua (V.R.); a_tol@ukr.net (A.T.); bugaich@iop.kiev.ua (S.B.); dmytrozhulai@gmail.com (D.Z.); klimush@iop.kiev.ua (G.K.); 2 V.I. Vernadsky Institute of General and Inorganic Chemistry of National Academy of Sciences of Ukraine, 03142 Kyiv, Ukraine; galynayarem@gmail.com (G.Y.); mirnaya@ionc.kiev.ua (T.M.); 3 Department of Physics and Engineering Physics, Central Connecticut State University, New Britain, CT 06050, USA

**Keywords:** ionic liquid crystals, nanocomposites, bimetallic core/shell nanoparticles, nonlinear optical properties

## Abstract

In this paper, we report the synthesis and characterization of unconventional nanocomposites made of bimetallic nanoparticles dispersed in a liquid crystal glass. Core-shell bimetallic nanoparticles (Ag/Au or Au/Ag) and Ag-Au bimetallic nanoalloys are synthesized using cadmium alkanoate glass-forming liquid crystals as nanoreactors. Optical spectra of the produced glassy nanocomposites exhibit a distinctive absorption peak due to a surface plasmon resonance. In addition, these unusual materials demonstrate a strong nonlinear–optical response probed by means of the Z-scan technique. The use of near-infrared (1064 nm) and visible (532 nm) nanosecond laser pulses reveal a variety of nonlinear–optical mechanisms that depend on the composition of the studied nanocomposites. Our results indicate that metal alkanoate-based glass-forming ionic liquid crystals with embedded plasmonic nanoparticles are promising, yet they are overlooked photonic nanomaterials suitable for optical and nonlinear-optical applications.

## 1. Introduction

The design and production of nanotechnology products relies on the synthesis and characterization of new nanomaterials [1] and references therein. As a rule, nanomaterials synthesized according to chemical, physical, and biological methods, are further dispersed in an appropriate liquid or solid matrix for their characterization and possible applications [1,2]. The need for a redispersion of nanomaterials implies additional cost and labor. Moreover, if nanoparticles are dispersed in liquid matrices, additional measures should be taken to reduce the aggregation of nanodopants and to preserve their functionalities [2]. Systematic studies of materials that could serve as a platform for the synthesis of nanoparticles and, simultaneously, act as their host matrix, are highly desirable. In this paper, we propose the use of metal alkanoate-based mesomorphic materials to achieve this goal.

Metal alkanoates are known for the versatility of condensed states of matter they can exhibit, including crystals, ionic liquid crystals, anisotropic glass, and liquids, to name a few [3,4,5]. Liquid crystal phases of metal alkanoates are of special interest because they can be utilized for a template synthesis of nanomaterials [6,7]. Typically, metal alkanoates form a smectic liquid crystal (LC) phase when heated to a temperature of 100–180 °C [8,9,10]. Because of their amphiphilic nature, metal alkanoates possess a useful property, in that they serve as universal solvent matrices for many organic and inorganic substances [8,9,10,11]. This fact enabled the use of metal alkanoate-based liquid crystal nanoreactors for the chemical synthesis of nanoparticles (NPs) of different origins. The synthesis technologies have been developed for semiconductor NPs (CdS, CdSe), metal NPs (Ag, Au) and core/shell NPs (Ag/CdS, Ag/CdSe, Au/CdSe. Ag/ZnS, Ag/ZnSe, CdSe/ZnS) [12,13,14]. An important feature of the proposed liquid crystal-aided nanotechnology that differs from conventional liquid crystal templating [7] is its simultaneous use of metal alkanoates as a nanoreactor and host matrix. The synthesized nanomaterials, instead of being washed out and redispersed in another material, remain uniformly distributed in a metal-alkanoate host/template. The produced NPs have a spherical shape (with the exception of CdSe NPs, which have the shape of plates) and a small dispersion of size, and are uniformly distributed within the volume of the metal alkanoate matrix [12,13,14]. 

Because mesomorphic phases of metal alkanoates exist at relatively elevated temperatures (100–180 °C), the produced liquid crystal nanocomposites containing nanoparticles seem be of little practical use. However, metal-alkanoate-based liquid crystals are excellent glass-forming materials [11]. Upon rapid cooling, they form an anisotropic liquid crystal glass that is stable at room temperature for years [11]. The glassy phase retains the same smectic structure as the LC phase, with NPs embedded inside. As a result, the unique properties of metal-alkanoates allow the production of nanocomposites made of anisotropic liquid crystal glass with embedded nanoparticles that are chemically synthesized in the liquid crystal phase of the same metal alkanoate template.

Simultaneous with the synthesis of new nanocomposites made of metal alkanoates and nanoparticles, systematic studies of their structural, optical, photovoltaic and nonlinear optical properties were carried out [11,12,13,14,15,16]. The obtained results unambiguously indicate that glass matrices of metal alkanoates can be used for the production of aggregation-free glass nanocomposites that allow optical and nonlinear-optical characterization of nanomaterials, along with the effects of guest–host interactions on the nonlinear-optical response of such materials. At the same time, metal-alkanoate-based glass nanocomposites exhibit all the properties of promising photonic nanomaterials that are suitable for many applications, including optical limiting [17,18,19,20,21], energy conversion and storage [22], nonlinear optical transformation of optical signals, ultrafast switching, and nonlinear integrated photonics [23,24]. With regards to the photonic applications of metal-alkanoate-based nanocomposites, plasmonic metal nanodopants are especially promising. The number of papers reporting optical and nonlinear-optical properties of plasmonic metal nanoparticles is constantly growing [25,26]. Bimetallic metal nanoparticles look especially promising due to their enhanced optical properties and a variety of photonic and biological applications [27,28,29]. 

Previous papers [11,12,13,14,15,16] have reported the technology and nonlinear-optical properties of metal-alkanoate-based glass containing silver and gold nanoparticles. This paper is a logical continuation of previous papers [11,12,13,14,15,16], with the goal of studying bimetallic plasmonic nanoparticles synthesized and dispersed in metal-alkanoate-based mesomorphic hosts/templates. More specifically, this work reports the preparation, structural, optical, and nonlinear optical characterization of cadmium octanoate nanocomposites with bimetallic core/shell NPs formed by the noble metals Ag and Au. The performed X-ray studies show that the bimetallic core/shell NPs can be formed in three different types: either Ag-core and Au-shell, or vice versa, Au-core and Ag-shell, or an Ag-Au bimetallic alloy, depending on molar concentration of starting compounds of tetrachloroaurate (III) and silver nitrate in chemical synthesis. In the optical spectrum, a pronounced band of surface plasmon resonance is observed for all three types of NPs in nanocomposites. Nonlinear optical studies were carried out using the Z-scan technique, with excitation at two wavelengths, the first harmonic, λ = 1064 nm and the second harmonic, λ = 532 nm, under the action of nanosecond laser pulses. We observed a change in the sign of the nonlinear refraction coefficient from a positive (self-focusing effect)—with excitation by the first harmonic—to a negative one (the self-defocusing effect) when excited by the second harmonic radiation for all samples. At the same time, nonlinear absorption exhibits the saturation absorption and reverse saturation absorption effects. The mechanisms of nonlinear optical effects in nanocomposites of cadmium octanoate with bimetallic NPs are discussed.

## 2. Materials Preparation: Chemical Synthesis of Bimetallic Nanoparticles in an Ionic Liquid Crystal Matrix

The synthesis of bimetallic nanoparticles was carried out in the thermotropic LC phase of cadmium octanoate (C_7_H_15_COO)_2_^−1^Cd^+2^, (abbreviated CdC8) by simultaneous chemical reduction of gold (Au^3+^) and silver (Ag^+^) cations from their compounds: tetrachloroaurate (III) (H[AuCl_4_] × 3H_2_O) and silver nitrate AgNO_3_, respectively [30]. The total concentration of NPs was 4% mol., or varied according to the formula [x Ag + (4 − x) Au] % mol. 

The structural characteristics of nanocomposites CdC8+Ag/Au with different molar ratios and combinations of gold and silver in NPs [Ag/Au (1:1); Ag/Au (3:1) and Au/Ag (3:1)] were studied using two methods: X-ray diffraction (XRD) and small-angle X-ray scattering (SAXS). It was found that by varying the composition of the reaction mixture or the duration of the synthesis, it is possible to obtain composites with NPs, such as bimetallic homogeneous or gradient alloys.

For optical and nonlinear optical measurements of nanocomposites, we have used cells obtained by the following methods. Solid powders of nanocomposites were placed between two glass substrates and heated to reach the LC state (100°–150 °C). After this, the cells were rapidly cooled to room temperature. This process resulted in the formation of glassy nanocomposite samples. The cell thickness was 30 µm.

## 3. X-ray Methods for Structural Characterization of Bimetallic NPs in Nanocomposites

The crystallographic parameters of the synthesized binary Ag/Au NPs were measured using an automatic X-ray diffractometer PROTO AXRD Benchtop X-ray diffractometer with a DECTRIS^®^ MYTHEN2 R 1D linear detector (Taylor, MI USA). Diffraction patterns were obtained in the wide angle range 2θ = 35 ÷ 135° (the following parameters were used: Cu Kα radiation (λ = 0.154 nm), voltage at the tube anode 30 kV, current 20 mA, Bragg-Brentano geometry, scanning mode θ/2θ, measurement step Δ2θ = 0.04°). Samples of nanocomposites heated to 110 °C were put on single-crystal silicon wafers in the form of a film of a thickness ~200 μm and rapidly cooled to room temperature. The CdC8 liquid crystal matrix acquired a glassy state, maintaining the ordering of the liquid crystals for a long time. PROTO PD Analysis v.1.7b software (Taylor, MI, USA) was used to process the measurement results. The size of bimetallic NPs in the CdC8 matrix was calculated using the Scherrer formula [31].

**Figure 1:**
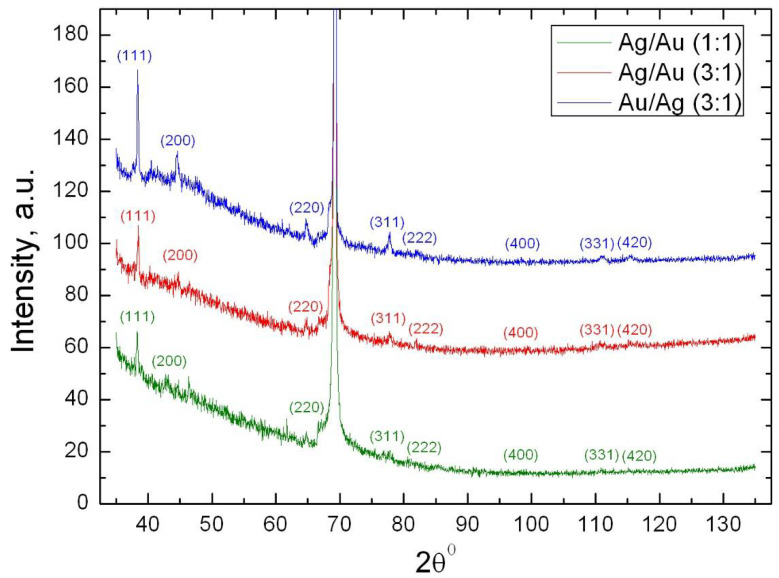
Powder X-ray diffraction patterns of three CdC8+Au/Ag nanocomposites with different molar ratios of Au and Ag.

Powders X-ray diffraction (XRD) of bimetallic nanocomposites CdC8+Ag/Au NPs with different molar ratio of silver and gold (namely Ag/Au (1:1), Ag/Au (3:1) and Au/Ag (3:1) NPs), are shown in Figure 1. In addition to the intense peak from the silicon substrate (crystallographic plane (400, 2θ = 69.20°), a set of diffraction maxima corresponding to a cubic face-centered lattice, the space group Fm-3m, a = b = c = 4.08 Å, is visible for all nanocomposites. The absence of additional crystal reflexes on the diffractograms indicates that the CdC8 matrix is in a glazed mesomorphic state. The ratios of the intensities of the indexed maxima and the parameters of the crystal lattice are very close to the corresponding values for the reference samples from the COD database: for Au (card 1100138) and for Ag (card 1100136). This indicates that the particles are likely to have a three-dimensional shape that is close to spherical. According to Scherrer’s formula, the average size of bimetallic nanoparticles was estimated; their size is about 20 nm for all three samples. It is much more difficult to interpret the internal structure of such nanosized bimetallic objects. The main issues that arise relate to the distribution of components within each particle (homogeneous or gradient alloy) and the outer face (predominant crystallographic planes).

The internal structure of synthesized NPs, as well as their shape and size, were also evaluated using small-angle X-ray scattering (SAXS). Nanocomposites in the form of a crystalline powder were put into thin-walled capillaries 0.7 mm in diameter and a wall thickness of 0.01 mm. Next, the samples were heated to a melting point (near 150 °C) for a more uniform filling of the capillaries. Further rapid cooling of the melt to room temperature led to the formation of highly stable glassy mesomorphic nanocomposites. X-ray studies were carried out using an automatic small-angle X-ray diffractometer based on an AMUR-1 goniometer with a slit collimator. A fine-focus X-ray tube with a copper anode (power 1.2 kW) and a fine focus 0.4 mm × 8 mm was used as a radiation source. The measurement conditions were as follows: U = 40 kV, I = 20 mA, the distance from the tube to the sample was 500 mm, and the distance from the sample to the detector was 350 mm. The intensity of scattered radiation was measured in the range of scanning angles 2θ = 0.10 − 3.75°, step Δ2θ = 0.01°, measurement time at each point t = 60 s. For better statistical reliability of the experiment, three separate measurements of the angular dependence of the intensity of X-rays radiation scattered by the sample and the background scattering of an empty capillary were performed, the results of which were averaged. The analysis of experimental data was carried out using the Monte-Carlo algorithm (program McSAS1.3.1 [32]).

**Figure 2:**
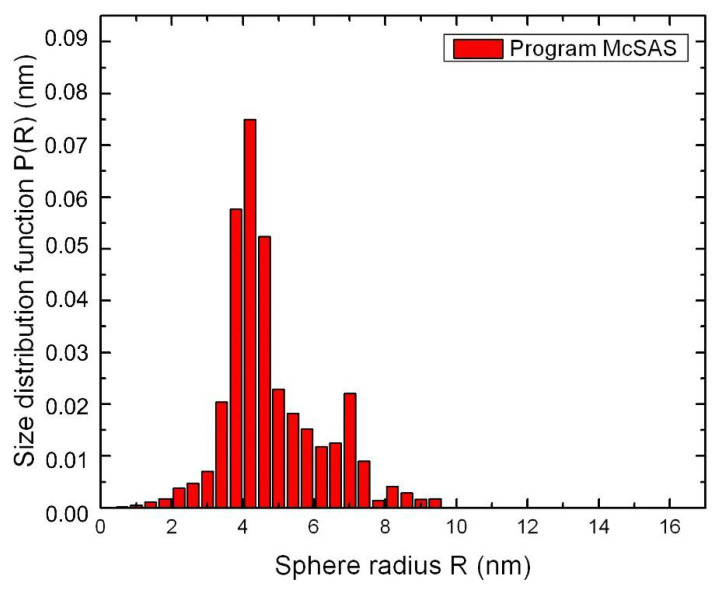
Size distribution function P(R) of nanoparticles Ag/Au (1:1) calculated by the Monte Carlo method.

The analysis of SAXS experimental data was carried out using the Monte-Carlo algorithm by McSAS1.3.1 program. Simulation conditions involve low concentrations of particles in an ionic LC matrix, a lack of interaction between them, a uniformity of shape and an inhomogeneity of their sizes. The method performed calculations in the approximate homogeneous spherical, inhomogeneous spherical and core-shell models. Figure 2 shows a size distribution function P(R) of nanoparticles Ag/Au (1:1). The most reliable results were obtained for the model of homogeneous spherical particles. This allows us to conclude that the studied samples are likely to contain stable hetero-nanoparticles, such as a metal alloy of homogeneous composition.

**Figure 3:**
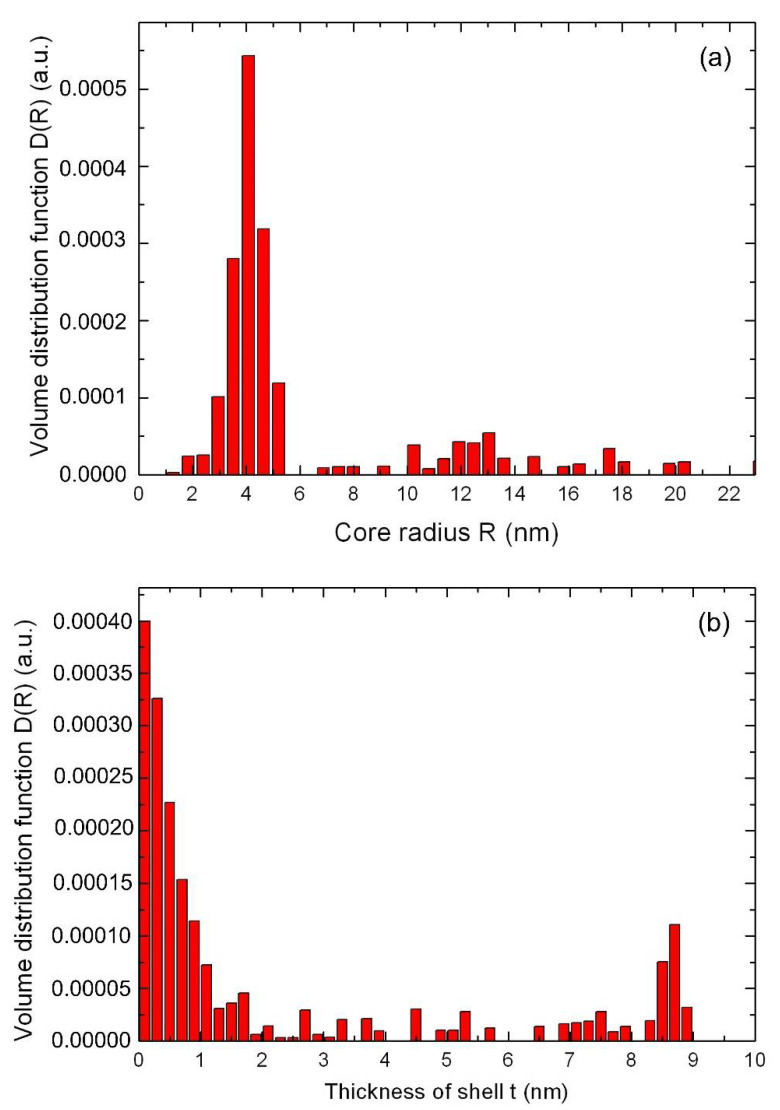
The size distribution function of Ag/Au (3:1) nanoparticle for the core (**a**), and the shell thickness distribution (**b**), calculated by the Monte-Carlo method in the core-shell model.

For a nanocomposite with a molar concentration ratio of nanoparticles of Ag/Au (3:1), according to small-angle X-ray scattering data, a different scenario is observed. For this sample, the core-shell structure is formed as follows. At the initial stage of chemical synthesis, due to rapid nucleation, bimetallic nanoparticles are formed from an Ag/Au alloy, after which, in the process of slow thermal diffusion, they are covered with a shell of gold atoms. As for the previous nanocomposite, the size distribution was calculated in the approximations of core-shell models. The results of the calculation by the Monte-Carlo method are obtained (Figure 3). As can be seen from Figure 3, in addition to nanoparticles with a core radius R~4.5 nm, a much larger radius R~12.5 nm was also observed in the nanocomposite. Such nanoparticles have an average radius of core Ag/Au NPs R~4.5 nm, covered by Au NPs with a shell thickness of t~8.5 nm. 

In a similar way, the core-shell structure can be found for nanocomposites with NPs Au/Ag (3:1).

## 4. Absorption Spectra of Nanocomposites

**Figure 4:**
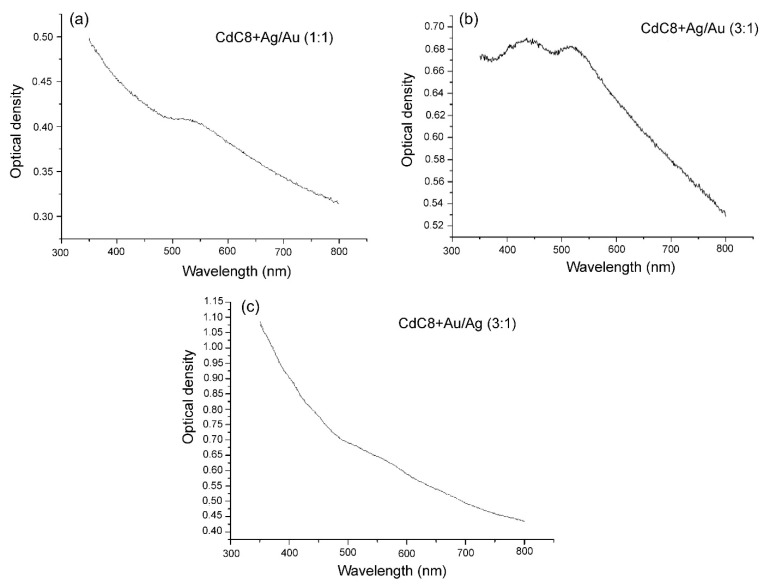
Absorption spectra of the composites CdC8+Ag/Au NPs of different structures. (**a**) The absorption band (SPR) of homogeneous alloy of Ag/Au (1:1) NPs of the CdC8 composites is shown. The absorption bands of core/shell NPs of Ag/Au (3:1) and of Au/Ag (3:1) in the CdC8 composites differ, which is seen from (**b**) and (**c**), respectively.

Optical measurements can also differentiate between core-shell and bimetallic structure of metal nanoparticles [33]. The absorption spectra of the glassy nanocomposites were measured using a universal automatic spectral complex KSVU-6 (LOMO). Studies of the absorption spectra of bimetallic NPs show that the weight ratio of silver and gold in NPs in the CdC8 composite leads to a change in the position of the maxima of the surface plasmon resonance (SPR) bands of NPs (see Figure 4).

Figure 4a shows an absorption band with a maximum at 525 nm, which corresponds to the SPR of homogeneous alloy of Ag/Au NPs. At same time, in Figure 4b, the absorption band exhibits two maxima: at 440 nm, which corresponds to the core of hybrid Ag/Au NPs, and a maximum at 520 nm, due to the Au-shell of the NPs. Figure 4c shows an absorption band with a maximum at 550 nm, which corresponds to the SPR of hybrid core-shell Au/Ag NPs and an absorption band due to the Ag-shell of NPs.

## 5. Nonlinear Optical Properties of Nanocomposites CdC8+Ag/Au NPs

The nonlinear optical characteristics have been studied for all three nanocomposites: CdC8+Ag/Au (1:1) NPs, CdC8+Ag/Au (3:1) NPs and CdC8+Au/Ag (3:1) NPs. A Q-switched Nd^3+^YAG laser (pulse duration: 9 ns, wavelength: 532 nm, repetition rate: 0.5 Hz) was used for the performed Z-scan experiment. Two wavelengths of laser excitation have been used: the fundamental model λ = 1064 nm and the second harmonic λ = 532 nm. The peak intensity I_0_ range was 11–12 MW/cm^2^. The incident Gaussian laser beam was focused using the lens, which had a focal length of F = 10 cm, and a beam waist of *w*_0_ = 54 μm for λ = 532 nm, and F = 13 cm and beam waist of *w*_0_ = 87 μm for λ = 1064 nm. During the Z-scan experiments, the samples were moved along the *z*-axis through the focusing area of the laser beam using a translation system. 

**Figure 5:**
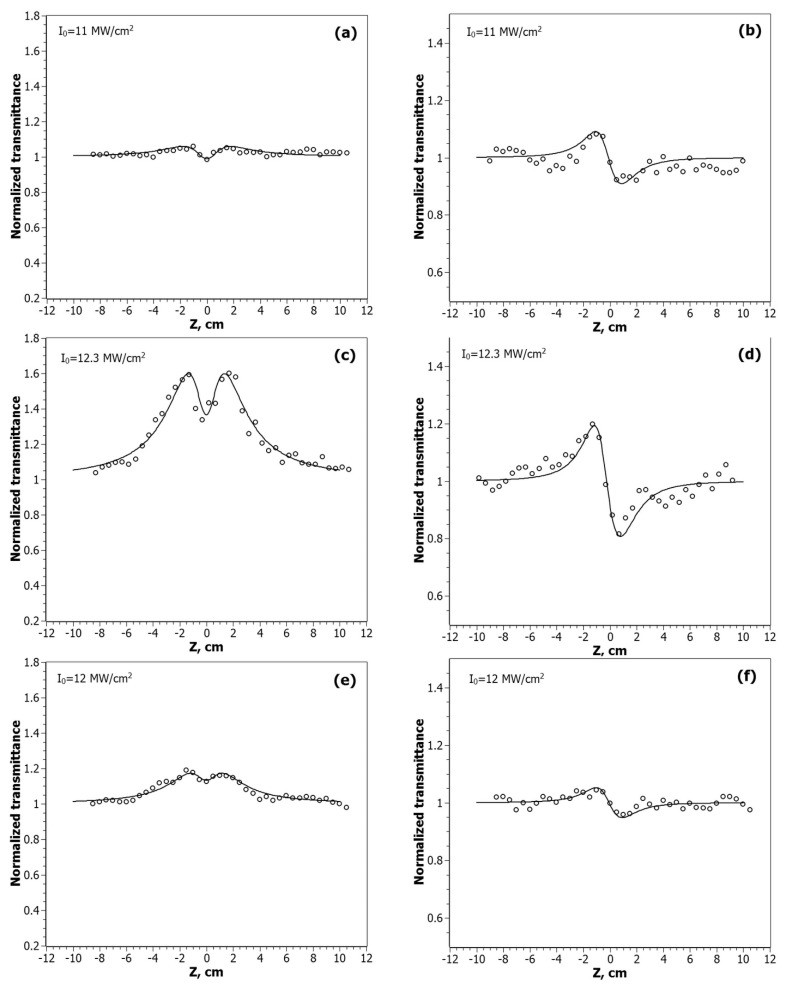
Z-scan profiles of the normalized transmittance measured for CdC8+Ag/Au NPs at the wavelength λ = 532 nm in the open-aperture (OA) scheme: CdC8+Ag/Au (1:1)—(**a**), CdC8+Ag/Au (3:1)—(**c**), CdC8+Au/Ag (3:1)—(**e**); and the closed-aperture (CA) scheme: CdC8+Ag/Au (1:1)—(**b**), CdC8+Ag/Au (3:1)—(**d**), CdC8+Au/Ag (3:1)—(**f**). Solid lines are theoretical fits of the experimental data.

**Figure 6:**
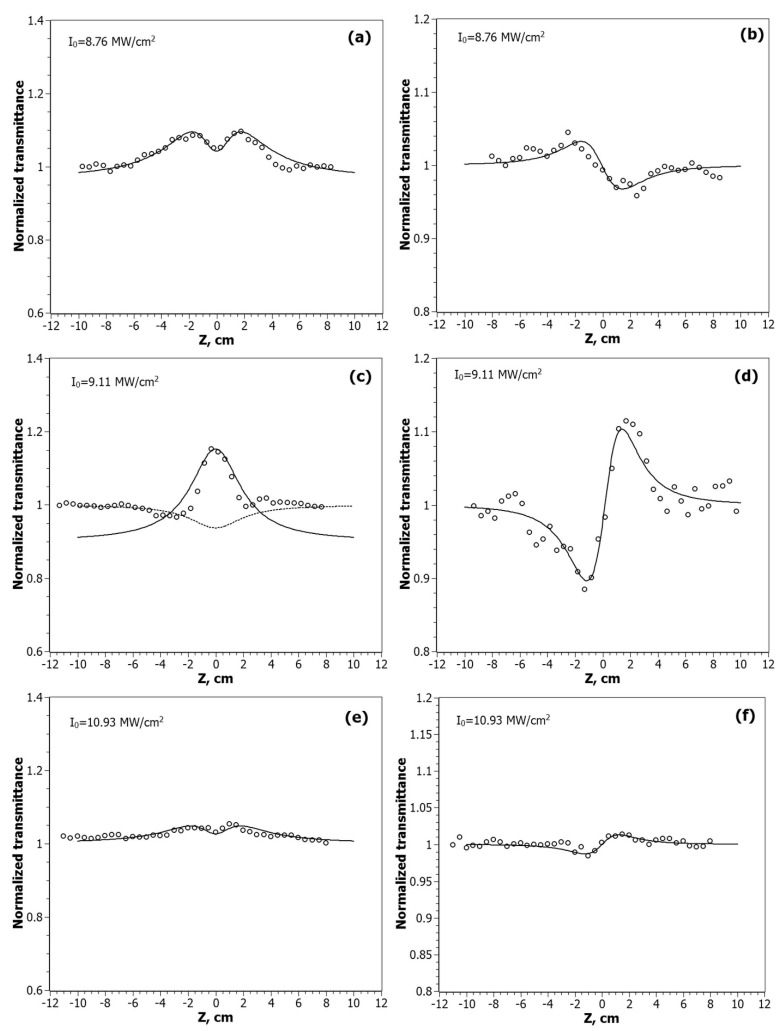
Z-scan profiles of the normalized transmittance measured for CdC8+Ag/Au NPs at the wavelength λ = 1064 nm in the OA scheme: CdC8+Ag/Au (1:1)—(**a**), CdC8+Ag/Au (3:1)—(**c**), CdC8+Au/Ag (3:1)—(**e**); and the CA scheme: CdC8+Ag/Au (1:1)—(**b**), CdC8+Ag/Au (3:1)—(**d**), CdC8+Au/Ag (3:1)—(**f**). Solid lines are theoretical fits of the experimental data.

The Z-scan curves of the nanocomposites at the λ = 532 nm are shown in Figure 5, and for λ = 1064 nm in Figure 6.

The nonlinear optical response of new nanocomposites can be associated with the SPR of bimetallic NPs at λ = 532 nm and SPR of two-photon excitation of NPs at λ = 1064 nm. The Z-scan scheme with an open aperture (OA) was used for the investigation of the nonlinear absorption process in the samples. The fitting of the obtained data was performed according to the equations [34,35]:

(1) $$T_{OA}\left(z\right)=\left[1-\frac{\alpha_{0}I_{s}L}{I_{s}+I_{0}/\left(1+z^{2}/z_{0}^{2}\right)}-\frac{\beta I_{0}L}{\left(1+z^{2}/z_{0}^{2}\right)}\right]/\left(1-\alpha_{0}L\right)$$

Here, *z*_0_ is the Rayleigh length, *I*_0_ is the peak intensity in the focal plane, *β* is the two-photon absorption (TPA) coefficient, *α*_0_ is the linear absorption coefficient, *L* is the thickness of the sample and *I_sat_* is the saturated intensity.

According to the obtained experimental data, the nature of the nonlinear absorption process varies depending on the position of the sample relative to the focus. Out of focus, there is a manifestation of saturated absorption (SA). When the sample approaches the focus, the process of reverse saturated absorption (RSA) begins to prevail. The intensity of these processes varies from sample to sample, depending on the ratio of gold and silver in the NPs. The Z-scan curve of CdC8 + Ag/Au (3:1) at λ = 1064 nm, shown in Figure 6c, was approximated by two equations. The upper curve is for the saturated absorption mechanism: T_SA_(*z*) = (1 + *I*_0_/*I_sat_*(1 + *x*^2^)), at *x* = *z*/*z*_0_; *I*_0_ is the peak intensity in the focal plane; *I_sat_* is the saturated intensity of the medium. The lower curve is for the mechanism of the reverse saturation absorption. With an increase in the input intensity *I*_0_, the SA begins to prevail over the RSA mechanism.

Several mechanisms are known for metallic nanoparticles that lead to nonlinear changes in light transmission: two- or multiphoton absorption [19], interband and intraband transitions [25,36,37,38,39], free carrier absorption, and nonlinear scattering. The contribution of each of them depends on the intensity, duration, and wavelength of the laser pulse. The SA mechanism, which we observe at lower intensity levels, is usually based on single-photon transitions between the valence band and the conduction band. At 532 nm (hν = 2.33 eV), single-photon interband transitions are possible for Au because its bandgap width E_g_~1.8 eV, but not for Ag, where E_g_~3.9 eV. At 532 nm, such processes (plasmon band bleaching) have already been observed for gold, silver, and their alloy nanoparticles [40,41,42,43]. With the sample approaching the focus, the intensity of the radiation incident on it increases, so that the mechanisms of a broadband-free carrier absorption and two-photon absorption (TPA) become dominant, which is what we have observed. The lowest values, *I_sat_* = 8 MW/cm^2^ and *β* = 2.5 × 10^−5^ at λ = 532 nm, were obtained by fitting the data found for nanocomposites with the NPs of the molar ratio Ag/Au (3:1); at λ = 1064 nm the lowest values *I_sat_* = 4.5 MW/cm^2^ and *β* = 1.03 × 10^−4^ for the nanocomposites with metal alloy NPs Ag/Au (1:1).

A closed aperture (CA) Z-scan scheme allows the sign and magnitude of the samples’ nonlinear refractive index to be determined. Approximation of the data for CA is done using the formula [44]:

(2) $$T_{CA}\left(z\right)=\frac{1}{1-\frac{4x}{{\left(1+x^{2}\right)}^{2}}\Delta \Phi_{0}+\frac{4}{{\left(1+x^{2}\right)}^{3}}\Delta \Phi_{0}^{2}}$$

where *x = z/z*_0_*,* Δ*Φ = kn*_2_*I*_0_*L*_eff_ is the on-axis phase shift at the focus due to nonlinear refraction, n_2_ is the nonlinear refractive index, and *L*_eff_ *=* [1 − exp(−*α*_0_*L*)]/*α*_0_ is the effective length of nonlinear medium.

The type of dependences obtained in CA experiments with nanocomposites indicates a negative sign of the nonlinear refractive index at λ = 532 nm, which corresponds to the defocusing process. As can be seen from Figure 5b,d,f, the magnitude of this process, as in the case of nonlinear absorption, depends on the molar ratio of gold and silver in the studied nanocomposites. It is known that the excitation of hot electrons and conduction electron intraband transitions are the main mechanisms that can contribute to the nonlinear refractive index change for metallic nanoparticles [45,46]. The laser wavelength is 532 nm, corresponding to the peak position of the SPR, which significantly increases this contribution. In addition, at nanosecond pulse durations, thermal effects will help make their contribution. The highest values of the real part of third-order nonlinear susceptibility, *Reχ*^(3)^ = −1.52 × 10^−8^ esu, are obtained for nanocomposites with the molar ratio Ag/Au = 3:1, in which structural studies have shown the presence of a gold shell around the Ag core. Since the thickness of this shell is small (t~8.5 nm), quantum-dimensional effects will play a significant role in enhancing nonlinear processes, as is shown, for example, in [47].

As for the CA measurements at λ = 1064 nm, the sign and value of the nonlinear refractive coefficient n_2_ depends both on the type of bimetallic NPs and on the light intensity *I*_0_. The negative sign of n_2_ for the alloy type NPs Ag/Au (1:1) (self-defocusing process) is changed to the positive sign (self-focusing) for core/shell NPs Ag/Au (3:1) and Au/Ag (3:1), as shown in Figure 6b,d,f. This indicates that the thermal mechanism of optical nonlinearity prevails for the nanocomposites with NPs Ag/Au (1:1). Furthermore, the value of n_2_ is higher for the NPs Ag/Au (3:1), and exhibits a focusing effect in a wide range of input intensities, and decreases with the increasing *I*_0_. At the same time, the positive sign of nanocomposites with NPs Au/Ag (3:1) shows the focusing process only at a high laser radiation intensity.

Nonlinear optical parameters measured for the samples for both wavelengths of the laser intensity are presented in Table 1. The obtained experimental data allow one to identify the optimal directions for the synthesis of ionic LC nanocomposites with specified parameters that are valuable for various applications.

The obtained values of nonlinear-optical parameters shown in Table 1 overlap with the data available in the literature for plasmonic nanoparticles dispersed in different matrices ($\left|\chi^{\left(3\right)}\;\right|\approx 10^{-8}-10^{-10}$ esu) [25,48,49] and references therein. Because the measured values of nonlinear-optical parameters reported in the literature can vary depending on the intensity of the laser beam, the pulse-width, nanodopant concentration, and the type of matrix, an exact comparison is not a trivial task. At the same time, the suitability of the designed materials for nonlinear-optical applications can be evaluated by calculating an appropriate figure of merit (FoM). The calculated FoM values are also shown in Table 1. Materials with their FoM values in the order of 1 or greater are considered promising nonlinear-optical materials. As can be seen from Table 1, CdC8 + Ag/Au (3:1) nanocomposite with an FoM value of 3.7 (λ = 1064 nm) and 0.8 (λ = 532 nm) is a promising material for nonlinear-optical applications which rely on third-order optical nonlinearities.

## 6. Conclusions

Embedding light-absorbing nanoparticles in transparent glass materials is a powerful approach for the creation of stable nanodispersions suitable for the development of commercial products. As a rule, the choice of available glass matrices is rather limited. Soda lime and borosilicate glasses are still the most common types of glassy materials actively utilized in the field of nanoscience and nanotechnology. Therefore, the design and characterization of new glassy materials containing nanodopants is highly desirable.

Ionic liquid crystals of metal alkanoates represent an overlooked class of glass-forming materials, which are promising for the design and study of the nonlinear-optical properties of nanomaterials. The thermotropic liquid crystal phase of cadmium alkanoate allows for the template chemical synthesis of various nanoparticles, including bimetallic nanoparticles. Thus, by varying the composition of the reaction mixtures and the duration of the synthesis of Ag/Au NPs in cadmium alkanoate, it is possible to produce nanoparticles such as bimetallic homogeneous or gradient alloys. 

In nanocomposite CdC8+Ag/Au (1:1) NPs, nanoparticles of the Ag/Au metal alloy type with an average radius of 6.2 nm were observed, according to the small-angle X-ray scattering method (Figure 1, Figure 2 and Figure 3). In the case of CdC8+Ag/Au (3:1) nanocomposites, NPs with an average radius of 13.0 nm, having an Ag core covered with an Au shell with a thickness of 8.5 nm, were formed. 

In this work, we experimentally observed the fast and large values of the nonlinear optical properties of these new glassy nanocomposites at an excitation of fundamental harmonic of the laser with a wavelength of 1064 nm and the second harmonic with a wavelength of 532 nm. By increasing the laser intensity, a transmittance of sample drops, which corresponds to the transition from the saturation absorption regime to the reverse saturation absorption regime. The refractive nonlinearity of all three samples has a negative sign at λ = 532 nm, at which point self-defocusing is observed, which at a nanosecond duration of laser pulses (9 ns) and at a high linear absorption indicates the dominant thermal mechanism of change in the nonlinear refractive index. At the same time, by switching to the fundamental mode of laser radiation with a wavelength of 1064 nm, a change in the sign of the nonlinear refractive index was detected: the nanocomposites can exhibit both self-defocusing and self-focusing effects, depending on the type of NPs (metallic alloy for NPs Ag/Au (1:1)) or core/shell NPs with an Ag core and an Au shell (Ag/Au (3:1)), or vice versa, Au/Ag (3:1), as shown in Table 1.

The most efficient self-focusing process was measured for CdC8+Ag/Au(3:1) nanocomposites in a wide range of input intensities. For CdC8+Au/Ag (3:1), the self-focusing process was observed only at high laser intensity. For CdC8+Ag/Au (1:1), a self-defocusing process was observed, which indicates the thermal mechanism of the nonlinear optical response. Our studies indicate an interplay between a number of different nonlinear-optical mechanisms at λ = 1064 nm due to two-photon absorption, such as the excitation of hot electrons and the intraband transition of conduction electrons, as well as the influence of quantum size effects due to the small size of the NPs shell. Thus, our results show that nanocomposites of cadmium alkanoate with bimetallic core/shell NPs can be used simultaneously for different types of modulation of laser signals depending on the wavelength. 

## Figures and Tables

**Table 1:** Nonlinear optical parameters of the three nanocomposites (CdC8+Ag/Au (1:1) NPs, CdC8+Ag/Au (3:1) NPs, and CdC8+Au/Ag (3:1) NPs).

Sample	λ, nm	*I*_0_, MW/cm^2^	*I*_s_, MW/cm^2^	n_2_, cm^2^/W	*Reχ*^(3)^, esu	*β*, cm/W	*Imχ*^(3)^, esu	*FoM* *
**CdC8 +Ag/Au (1:1)**	1064	8.76	4.5	−1.13 × 10^−9^	−9.88 × 10^−9^	1.03 × 10^−4^	4.97 × 10^−8^	0.06
**CdC8 +Ag/Au (3:1)**	1064	9.11	36	6.56 × 10^−10^	2.8 × 10^−8^	0.5 × 10^−5^	2.41 × 10^−9^	3.7
**CdC8 +Au/Ag (3:1)**	1064	10.93	24	4.77 × 10^−11^	2.04 × 10^−9^	-	-	-
**CdC8 +Ag/Au (1:1)**	532	11	17	−2.39 × 10^−10^	−1.02 × 10^−8^	3.7 × 10^−5^	8.92 × 10^−9^	0.36
**CdC8 +Ag/Au (3:1)**	532	12.5	8	−3.55 × 10^−10^	−1.52 × 10^−8^	2.5 × 10^−5^	6.03 × 10^−9^	0.80
**CdC8 +Au/Ag (3:1)**	532	12	13	−1.04 × 10^−10^	−0.44 × 10^−8^	2.95 × 10^−5^	7.11 × 10^−9^	0.20

* $FoM=\frac{1}{\pi }\left|\frac{Re\chi^{\left(3\right)}}{Im\chi^{\left(3\right)}}\right|$.

## Data Availability

All data that support the findings of this study are included within the article.
